# Persistence of Lipoproteins and Cholesterol Alterations after Sepsis: Implication for Atherosclerosis Progression

**DOI:** 10.3390/ijms221910517

**Published:** 2021-09-29

**Authors:** Krzysztof Laudanski

**Affiliations:** 1 Department of Anesthesiology and Critical Care, University of Pennsylvania, Philadelphia, PA 19104, USA; klaudanskil@gmail.com; Tel.: +1-215-662-8200; 2 Department of Neurology, University of Pennsylvania, Philadelphia, PA 19104, USA; 3 Leonard Davis Institute of Healthcare Economics, Philadelphia, PA 19104, USA

**Keywords:** sepsis, septic shock, lipid profile, apolipoproteins, atherosclerosis, neurodegeneration, long-term outcome, allostasis

## Abstract

(1) Background: Sepsis is one of the most common critical care illnesses with increasing survivorship. The quality of life in sepsis survivors is adversely affected by several co-morbidities, including increased incidence of dementia, stroke, cardiac disease and at least temporary deterioration in cognitive dysfunction. One of the potential explanations for their progression is the persistence of lipid profile abnormalities induced during acute sepsis into recovery, resulting in acceleration of atherosclerosis. (2) Methods: This is a targeted review of the abnormalities in the long-term lipid profile abnormalities after sepsis; (3) Results: There is a well-established body of evidence demonstrating acute alteration in lipid profile (HDL-c ↓↓, LDL-C -c ↓↓). In contrast, a limited number of studies demonstrated depression of HDL-c levels with a concomitant increase in LDL-C -c in the wake of sepsis. VLDL-C -c and Lp(a) remained unaltered in few studies as well. Apolipoprotein A1 was altered in survivors suggesting abnormalities in lipoprotein metabolism concomitant to overall lipoprotein abnormalities. However, most of the studies were limited to a four-month follow-up and patient groups were relatively small. Only one study looked at the atherosclerosis progression in sepsis survivors using clinical correlates, demonstrating an acceleration of plaque formation in the aorta, and a large metanalysis suggested an increase in the risk of stroke or acute coronary event between 3% to 9% in sepsis survivors. (4) Conclusions: The limited evidence suggests an emergence and persistence of the proatherogenic lipid profile in sepsis survivors that potentially contributes, along with other factors, to the clinical sequel of atherosclerosis.

## 1. Introduction

Sepsis is one of the leading causes of mortality, affecting a vast stratum of society with increasing prevalence due to a multitude of factors [1,2]. Improvement in diagnosis, treatment, and management of sepsis has led to short-term mortality improvement with, therefore, more and more survivors suffering from the long-term consequences of sepsis [3,4,5,6,7]. Even more significantly, the COVID-19 (SARS-CoV2 disease of 2019) pandemic has continued to affect several millions of people, with at least 5% of victims suffering from severe critical illness, and even more from illness, with potential long-term consequences [8]. This further increases the incidence of individuals suffering from prolonged consequences of sepsis. Furthermore, sepsis survivors are frequently at higher risk of secondary sepsis and infection, potentially worsening the long-term consequences of the first episode [7,9,10]. Therefore, it is not surprising that sepsis survivorship is of concern and is referred to as the “silent epidemic” [1].

Sepsis is a complex and multifaceted disease. It is not a singular nosological entity, but should be considered several diseases defined as one, due to our current clinical inability to provide a more accurate gestalt. The Centre for Disease Control calls sepsis the *“…body’s extreme response to an infection, … a life-threatening medical emergency. … and happens when an infection you already have triggers a chain reaction throughout your body”* [2]. Sepsis Alliance describes sepsis as *“your body’s overactive and toxic response to an infection”* [11]. Finally, the Society for Critical Care Medicine terms sepsis *“as life-threatening organ dysfunction due to a dysregulated host response to infection”* [12]. The common motif of these definitions is a dysregulated response to the presence of a pathogen. Most of the attention has been paid to the initial, or early, part of sepsis. Teleologically, this approach is well-grounded as only survivors will suffer from medium- and long-term sepsis consequences. It is well-established that untreated initial sepsis results in clinical demise, due to the overwhelming immune response or by unimpeded pathogen growth that eliminates the host. Response to early sepsis is also one of the major determinants of the evolution of post-septic allostasis [13,14,15]. Dysregulation of this response is at the heart sepsis pathology, as the host’s response must be swift, adapt quickly, and must precisely focus the response on the invading pathogen, while minimizing organ damage due to the pathogen’s virulence, and collateral damage from the initial immune response.

This somewhat simplistic concept was enriched recently by the appreciation of several other dynamic states between host and invading pathogen. One of the most common, yet frequently overlooked, states is bacteraemia, in which bacterial pathogens are detected in the blood stream with a perceivable response [16,17]. Some would suggest that this is an adaptive response, even though bacteraemia represents a pathological state according to the homeostasis concept [18,19,20]. Therefore, the emergence of a new state of allostasis needs to be appreciated, while the question of how long it takes—under optimal conditions—for the homeostasis to be restored to pre-insult state may not be applicable in sepsis survivors.

Homeostasis is the basic medical concept that the body is trying to maintain a pre-determined set of parameters within certain ranges and that any deviation needs to be counteracted to restore the pre-insult status quo [21,22]. However, since sepsis is an example of a dysregulated response, how long, and if at all, can homeostasis be recovered by survivors is an open question. The epidemiological data supports that this is a prolonged process, and that the newly emerged balance is allostatic [23]. This appreciation of incomplete recovery is somewhat new thinking in the medical community.

The long-term psychological, economical, and societal consequences of sepsis are of increased appreciation among the medical community and are reflected in legislative and reimbursement incentives [1,24,25,26]. Despite the numerous efforts to improve the well-being of sepsis survivors, these individuals are part of a hidden epidemic. The framework of post-intensive care syndrome (PICS) has focused mostly on rehabilitation and support, while little research has explored the potential to avert sepsis’s long-term effects and to regenerate baseline health by intervention, utilizing molecular biology techniques [24,27,28]. Alternatively, the healthcare stakeholders could choose to view their early intervention as having a significant long-term impact rather than focusing solely on short-term gains.

It has been known for some time that survivors of critical care illness suffer from post-sepsis syndrome, including anergy, hyperreactive airway, cognitive decline, sleep problems, mobility deterioration, and progressive organ dysfunction [5,29,30,31,32]. Some of these morbidities have been observed in the COVID-19 “long haulers”, or individuals recovering from influenza, as a part of the post-viral syndrome [33,34,35]. The emergence and persistence of post-septic abnormalities strongly suggest that sepsis sets processes in motion that perpetuate independently of the initial insult. Consequently, not homeostasis, but the allostatic balance applies much more adequately to describe the survivors’ health status. Homeostasis assumes a return to optimal physiology. This term may be applicable to some “seemingly recovered” sepsis survivors. However, these individuals are unlikely to be in the prior state of health even though they do not suffer from overt chronic disease. However, their post-sepsis allostasis is not part of the natural senescence process. In fact, several authors provide significant evidence that sepsis results in cellular reprogramming that make it impossible to return to pre-insult homeostasis [20,36,37,38,39,40,41].

Among many long-term mechanisms that sustain the post-septic sequela, metabolic and immune system abnormalities are the two most commonly suggested [8,26,29,30,42]. Interestingly, both mechanisms intersect profoundly with pathways of lipid metabolism in short, medium, and long-term time frames. Conversely, the lipid profile may be a pivotal component of the several co-morbidities typically seen in sepsis survivors. Though direct links between lipid profile abnormalities and post-septic conditions are not studied in-depth, cholesterol metabolism is critical in the natural history of dementia, cognitive decline, and cardiovascular system performance [43,44,45]. All of these conditions are often seen in the aftermath of sepsis [10,29,30,32,33,46]. A large meta-analysis looking at the long-term complication of sepsis demonstrated that the risk of stroke, myocardial infarction, and congestive heart failure increased by 3% to 9% in sepsis survivors, but the data were of low certainty [47]. The pooled risk from 29 highly heterogeneous studies suggested, at a minimum, that acquired abnormalities in lipid profile post-sepsis may be one of the underlying mechanisms of attained and permanent post-sepsis allostasis balance.

During acute septic episodes, components of cholesterol metabolism are critical in response to infection by moderating the immunological response [48,49,50,51,52]. Lipoproteins modulate immunological function by removing inflammatory markers and affecting leukocyte and endothelial performance. Therefore, acute lipid abnormalities in sepsis are likely to significantly affect the degree of potential organ damage and cellular leukocyte reprogramming, which are pivotal in determining the extent of recovery or progression of deterioration in homeostasis [14,36,37,38,39]. Secondary, if the lipid abnormalities, or dyslipidaemias, persist after resolution of the acute septic phase, they may play a key role in the progression of atherosclerosis-related illnesses and diminish the response to subsequent infections after an acute episode [53,54,55,56,57]. However, there are some gaps in the knowledge demonstrating how these dyslipidaemias may translate into long-term health maintenance.

This is a review of the current state of knowledge aimed at identifying potential abnormalities in lipoproteins and cholesterol as a potential culprit for the emergence of post-septic co-morbidities and long-term mortality due to atherosclerosis acceleration [21,22,23]. In this review, we did not include other important components of atherosclerosis (macrophages, endothelial function, metabolic syndrome) [54,55,57,58,59,60,61]. The review focuses on clinical situations consistent with commonly defined viral, bacterial or protozoal infections consistent with sepsis or bacteraemia definitions [2,12,25]. The review does not cover chronic inflammatory processes (chronic hepatitis, acquired immunodeficiency syndrome (AIDS), and others) as they represent different conditions and deserve dedicated reviews of their own.

## 2. Lipid Metabolism under Nominal Conditions

### 2.1. Cholesterol and Lipoproteins—Function and Properties

A lipid profile generally refers to direct and indirect estimates of total cholesterol, high-density lipoprotein cholesterol (HDL-c), and triglycerides (TG), allowing for calculation of cholesterol associated with very low-density lipoproteins (VLDL-c), and low-density lipoprotein cholesterol (LDL-c) [62].

Lipids and cholesterol are typically transported as lipoproteins, assemblies consisting of triglyceride and cholesterol in the centre surrounded by a phospholipid shell with apolipoprotein embedded in it. Phospholipids provide an interface that allows for hydrophobic lipid to enter the centre of the lipoprotein and ensure the basic function of lipoproteins—lipid transportation through the hydrophilic environment of blood [63]. Detergent-like features enabling hydrophobic material to enter into the micelle of lipoproteins are provided by apolipoproteins (Apo). Apo is also critical in regulating the turnover of lipoproteins through different cells and organs [52,64,65,66,67,68]. Several apolipoprotein types exist with diverse physiological functions and corresponding roles in different diseases (Table 1).

Most of the research denoting the role of apolipoproteins is focused on their role in genetic-driven abnormalities, which are inherited traits [44,82]. These genetic predispositions are pivotal in determining several atherosclerotic processes, but they are not modifiable. However, apolipoproteins can be chemically modified over their lifespan (oxidized, acetylated, others) with a profound effect on their function [59,120,121]. These modifications interrupt the normal metabolism of the lipids, leading to the acceleration of related abnormalities. In contrast to genetic traits, chemical modifications or changes in the composition of apolipoproteins represent acquired features, while genetic-driven abnormalities predispose an individual for life [44,70,72,83,86,87]. Thus, the former mechanism provides a potential option of how inflammation can affect cholesterol metabolism over the lifespan. Additionally, metabolic abnormalities can affect lipoprotein composition.

### 2.2. Lipid Metabolism under Physiological Conditions

Under nominal conditions, the metabolism of cholesterol is relatively straightforward and consists of endogenous and exogenous sources. Most of the cholesterol is produced in the liver (~70%), while the exogenous supply is delivered with food (~30%). The mevalonate pathway synthesizes the endogenous cholesterol. The rate-limiting part of that pathway is synthesis of mevalonate by β-Hydroxy β-methylglutaryl-CoA (HMG-CoA) reductase [122]. The reductase is also the primary point of interference for a statin. During stress, de novo metabolism in the liver mostly switches to the exogenous supply, as manufacturing cholesterol is energy expensive [122]. In the exogenous supply of cholesterol, lipid particles are transported from the intestine as the chylomicrons through the body. The liver is the main regulator that captures the excessive chylomicron, or their remnants, via receptors recognizing ApoE, LDL-c or other specialized receptors.

Irrespective of the source of the cholesterol, the liver repacks the excess cholesterol into VLDL-c and releases them into the bloodstream. Endothelial lipoprotein lipase (LPL) catalyses triglyceride and facilitates receptor-mediated cholesterol uptake, relying on ApoE and ApoB100 [123,124]. This allows endothelium to be supplied with triglycerides and, to a very limited degree, with cholesterol. At the same time, VLDL-c is being converted into IDL. LDL-c has several controlling mechanisms that are part of metabolic homeostasis and are tightly linked to glucose metabolism. Intermittent density lipoprotein (IDL-c) can then be metabolized by the liver or can continue to shed triglycerides during their transition through the body. In both cases, LDL-c emerges as the main cholesterol transporting molecule, a process allowing the incorporation of cholesterol in the cell wall.

Accumulation of LDL-c is a negative signal for de novo cholesterol synthesis [122]. Dysfunction of this negative feedback mechanism results in abnormal uncontrolled activation of cholesterol synthesis. This excess of cholesterol is not unfavourable per se, but it can be chemically modified (oxidized) by exposure to an extracellular environment, altering the normal cholesterol pathway metabolism further via several HMG-CoA reductase independent mechanisms [125,126]. These mechanisms may be particularly pronounced in high oxidized states, resulting in lipid alterations (peroxidization, acetylation, carbonation) [126]. In any case, macrophages pick up modified, or excessive, LDL-c with subsequent functional transformations (into foamy cells, atypical monocyte, or M2 type macrophages) followed by their accumulation in the endothelium, astrocyte, and platelets. This accumulation of transformed monocytes leads to several abnormalities that may trigger several illnesses related to accelerated atherosclerosis or direct toxicity (Table 1) [126,127,128,129]. The excess of cholesterol can be evacuated and transported to the liver via HDL-c, providing the mechanism to reduce cholesterol load.

Even from this extremely brief description, it is apparent that cholesterol metabolism is a dynamic balance between several components and is intertwined with metabolic regulation, which is described elsewhere [128]. Furthermore, metabolism of cholesterol in different tissues—which are involved in cholesterol turnover—may be affected differently in a variety of tissues by the inflammatory process, hormonal abnormalities, or metabolic abnormalities [122,130,131] Apolipoproteins are the key determinants of turnover and functional processing as they serve as the trafficking points for determining cholesterol processing. Finally, lipid metabolism requires unaltered lipoprotein structure since chemical modification results in an abnormal turnover.

### 2.3. Role of the Lipids in Homeostasis

Lipids, and cholesterol, have several critical functions. First, their optimal level is tightly regulated as hypolipoproteinaemia equals a severe disturbance in homeostasis, while hyperlipoproteinemia is critical in fuelling several pathological processes.

Cholesterol is a critical component of the cell wall. Its highly hydrophobic structure, electrical impedance, and rigidity are critical for the mechanical and electrical integrity of the cell wall [63,132]. Several critical signalling molecules utilize cholesterol and lipid as the backbone of their structures. So-called lipid-soluble hormones are derivatives of lipids [133]. Prostaglandin and thromboxane are examples of diverse groups of biologically active molecules originating from lipids critical in several areas of health [133]. However, the most important pathological role for cholesterol is related to its immunological properties. In addition to being a building block of prostaglandin and thromboxane, lipids are critical in scavenging lipopolysaccharide from blood to limit the immunological response. They effectively translocate lipopolysaccharide (LPS) from the blood into the liver to limit duration of exposure of the body to toll-like receptor (TLR) stimulation [134,135,136,137,138,139]. Removal of LPS is one of the most potent immune responses during the acute phase of sepsis and determines long-term outcomes. HDL-c also binds to lipoteichoic acid (LTA), affecting inflammatory responses mediated through TLR2 [140]. HDL-c also plays an important role in modulating viral responses via separate mechanisms, but the clinical mechanism is pathogen-dependent [141]. HDL-c can facilitate cellular entry of hepatitis C, Dengue, or SARS-CoV-2 virus [142,143,144]. On the other hand, HDL-c attenuates inflammation secondary to influenza exposure in the lungs by modulating macrophage responses and trafficking [145].

The beneficial role of the lipoproteins is most likely reflected in the clinical observation demonstrating that diminished serum levels of LDL-c and HDL-c highly correlate with short-term mortality (defined as less than 28 days) in sepsis. Sepsis-concomitant hypolipoproteinaemia could be secondary to increased consumption, exaggerated catabolism, starvation, and liver dysfunction, but it triggers a uniform depletion of HDL-c and LDL-c. The subsequent lack of protective HDL-c and LDL-c may emerge as an independent feature in sepsis [137,139,146]. Thus, the depression of the lipoprotein during acute phase infection may have far-reaching effects. In general, the duration and character of the initial response of the sepsis determines long-term outcomes, even though lipoproteins are not yet implicated in mechanisms [38,39,46,147,148]. However, two mechanisms can be suggested about potential influence. First, exaggerated immune system response due to the insufficient protection offered by lipoproteins may lead to immunological exhaustion and increased susceptibility to secondary infection [40,149,150]. Additionally, reprogramming of the leukocytes during the initial sepsis is pivotal for the long-term performance of the immune system. Consequently, abnormally low HDL-c and LDL-c levels may affect outcomes indirectly. Some suggest supplementation of the lipoproteins in sepsis to achieve an immunomodulatory effect [151]. It is also worth underscoring that most of the studies suggested the beneficial role of LDL-c in moderating immune response, while the role of HDL-c is much more ambivalent.

On the other side, hypercholesterolemia results in the acceleration of atherosclerosis with a long-term effect on several aspects of health [56,60,128,152]. This acceleration of atherosclerosis, secondary to dyslipidaemias, must be put in the special context in sepsis, as the illness itself triggers several pro-atherogenic conditions [54,57]. Endothelial inflammation, a free radical environment, the emergence of atypically activated monocytes, impaired glucose metabolism, increase serum C-reactive protein, and abnormalities in homocysteine metabolism are the main factors contributing to the progression of atherosclerosis during and after sepsis [58,61,153,154,155]. Here, we will focus on the effect of lipid metabolism abnormalities on the progression of atherosclerosis only. This review will not cover the effect of acute lipid abnormalities on cell reprogramming during the initial response to sepsis.

## 3. Lipid Metabolism during Acute Sepsis

### 3.1. Current Understanding of Acute Sepsis and Post-Sepsis Milieu

Challenge by pathogens triggers a response from the immune system aimed initially at *curbing* the initial growth of the pathogen, followed by transformation into *precise* elimination and acquisition of long-term immunity, while reconstituting damaged organs [30,57,58,148]. This process relies on several innate and acquired immunity components to harmonize complex and carefully choreographed, yet highly redundant processes. Thus, it is not surprising that the immune system and lipid profile are mutually intertwined during the natural evolution of the host’s response to invading pathogens.

**Figure 1:**
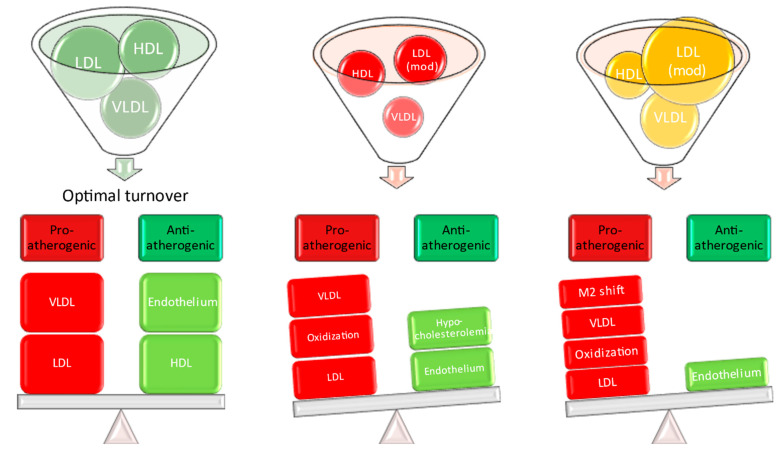
Acute sepsis causes profound disturbances in lipid profile while subsequent recovery has distinctive yet also abnormal profile.

Sepsis defines a situation when this precise process deviates from the optimal trajectory, failing to fulfil the objectives mentioned above. In addition, metabolic reprogramming, the free radical environment, and liver dysfunction facilitate the interaction between the immune system and lipid metabolism [20,46,126]. Consequently, it is possible that during and after sepsis, a lipid profile acquires new features which are distinct from the preceding state of health and different from those observed in the acute phase (Figure 1). 

Furthermore, if these conditions persist over a long period, even small deviations may lead to significant impacts [129]. Both sepsis and atherosclerosis are illnesses in which optimal function is lost or altered.

### 3.2. Lipid Metabolism during Acute Sepsis

HDL-c and LDL-c levels are almost uniformly depressed proportionally to sepsis’s severity and are accompanied by the oxidation of LDL-c (ox-LDL-c) [16,41,154,156,157,158,159,160,161,162,163,164]. Oxidation of HDL-c is more pronounced in older patients due to the small footprint of antioxidants [165]. Similar alterations in the lipid profile are apparent in viral and protozoan-driven sepsis as well [164,166,167,168]. Some studies quantified quite precisely that a serum level of HDL-c below 25 mg/dL is the threshold of dramatically increased risk for multiorgan failure in sepsis [169,170]. These changes are typical for both sepsis and bacteraemia but not trauma, suggesting specificity of hypocholesterolaemia for infectious processes [16,171]. Having a lower pre-existing LDL-c level results in an even less favourable outcome in pneumonia [172]. This is an interesting finding as taking LDL-c lowering medication, statin, resulted in a more beneficial outcome [173]. One potential explanation for the beneficial effect of statin (apart from the pleiotropic effect) is that low-grade inflammation results in a persistent increase in LDL-c, not depression [174]. Statins also have a complex, yet not fully accounted for, pleiotropic effect. Serum VLDL-c levels were increased during the acute illness [175]. The components of more advanced parts of the lipogram were not studied in depth, except for the demonstration that ApoA-1, which is depressed during the sepsis episode [138,156,157]. The abnormalities of several other components of lipid profile are being studied right now using lipidomic techniques. In general, these studies confirm prior observation of the decline of LDL-c and HDL-c during acute sepsis, but during acute sepsis, but did not confirm the correlation between clinical outcome and the level of decline [135,164].

### 3.3. The Biological Significance of Lipid Metabolism during Acute Sepsis

The level of lipid abnormalities is proportional to the severity of illness and is the hallmark of survival vs. demise [16,41,154,156,157,159,160,161,162,163]. However, one study did not demonstrate the relationship between LDL-c and HDL-c and mortality in COVID-19, suggesting a potential pathogen-specific response [164].

Lipoproteins have important immunological functions, which includes scavenging inflammatory mediators and transporting them in the liver [135,153]. Removing the bacterial pathogen-associated molecular patterns from the bloodstream significantly limits inflammation and cellular reprogramming [20,24,36,37,39,46]. Consequently, this may be the primary mechanism of lipoprotein by which they impact the natural history of sepsis and long-term sequela. Animal studies demonstrated some benefits of artificial lipid-like compounds to limit inflammation, translocate leukocytes into end organs, and enhance clearance of the bacterial load [176]. Lipids also have a critical effect on monocyte performance by altering their differentiation [177,178]. Even more importantly, ingestion of ox-LDL-c increases apoptosis, activation of monocyte, and mitochondrial dysfunction [179,180]. The endothelium responds to ox-LDL-c in a similar way, leading to potential damage [179]. OxLDL-c can also trigger the differentiated T cell immune response via uptake and stimulation of dendritic cells, but this mechanism’s specific role in promoting atherosclerosis needs to be elucidated [181].

Apoptosis is one of the hallmarks of poorly regulated and morbid sepsis during the acute and recovery phase. Apoptosis can directly damage the endothelium, making it prone to atherosclerosis injury. Concomitant aberration in monocyte differentiation can propel atherosclerosis by activating one of pro-atherosclerotic subpopulations of monocytes: atypical monocyte 2 (M2) or foamy cells [33,39,41,54,56,57,58,66,153,182,183,184]. M2 represents an atypical, activated monocyte that produces a significant amount of monocyte-colony stimulating factor (M-CSF) and free radicals, while not being able to mount an effective response to invading pathogens. These cells are frequently implicated in the emergence of atherosclerosis and immunosuppression [185]. Ingestion of apoptotic cells, commonplace in sepsis, and abnormal cholesterol metabolism is often linked to their emergence [186,187]. They can also persist for a prolonged period, resulting in potential maintenance of pro-atherosclerotic conditions.

It is important to point out that hyperlipoproteinemia during sepsis contributes to mortality and it is not an epiphenomenon. This is an important consideration as an alteration in lipids may be secondary to malnutrition, intestinal absorption in the gut, hormonal changes, or impaired synthetic function of the liver [46,134,150,153,188]. All these conditions are commonplace in sepsis, but abnormalities in lipids are secondary to increased turnover and exposure to oxidizing conditions, resulting in lipoprotein chemical modifications with profound effects on immune system performance [8,56,128,156,160].

## 4. Lipid Metabolism during in Sepsis Survivors

### 4.1. Lipid Metabolism during Recovery from Sepsis

Few studies of the lipid profile extended the observation period past the acute phase of sepsis. Van Leuven et al. demonstrated the suppression of HDL-c and LDL-c -c at four weeks after the onset of sepsis in human survivors [136]. A modification accompanied the HDL-c composition decreased with increased serum amyloid A concentration during recovery [136]. This alteration in HDL-c composition is linked to increased platelet levels and monocyte activation, potentially accelerating atherosclerosis [123,139]. Amyloid A may be implicated in the exacerbation of diastolic heart dysfunction. Tanaka et al. found a post-sepsis decrease in LDL-c, HDL-c, and cholesterol 28 days post-admission [189]. Similar data are seen in COVID-19, but the study was limited to 28 days and the onset of illness is often difficult to ascertain [164].

In both cases, the unresolved question is the impact of the synthetic function of the liver, and nutritional status in general, both possibly serving as confounding factors [32,134,153]. It is also unclear if sepsis and post-septic inflammation was fully resolved in these patients. In all presented studies, the effect of sepsis on the acceleration of long-term lipid profiles was not the primary hypothesis. The scant data repetitively and independently corroborate that even after 30 days post-sepsis, persistent, qualitative, and quantitative changes existed in the lipoprotein profile in survivors. Only a few studies extended the observation period past 28 days. Most longitudinal data were supplied from a patient with resolved Brucella infection four months after resolution, demonstrating elevated levels of LDL-c, HDL-c and cholesterol [183]. The level of oxidized LDL-c was identical during recovery and acute Brucellosis. Concomitantly, ApoAI, ApoB, and ApoCIII were elevated as well. Notably, all these apolipoproteins play a critical role in the emergence of Alzheimer’s disease and coronary artery disease [53,66,71,83,87,89,119]. VLDL-c and Lp(a) were unchanged on follow-up, suggesting high specificity of the post-septic changes [183]. In another study, patients recovering from acute Epstein-Barr infection were followed. However, in these patients, the serum level of LDL-c and their size, HDL-c, apoB, apoCIII, and Lp(a) levels were lower during acute infection., but fully recovered as observed in healthy controls [182]. Two studies addressed the lipid profile after recovery from leptospirosis and leishmaniosis [190,191]. The consistent finding was a decrease in HDL-c at four months. Additionally, apoAI was decreased at four months after visceral leishmaniosis but not leptospirosis [191].

The existing studies need to be analysed cautiously. First, most of the studies involved small numbers of individuals over a short period. It is difficult to call these studies long-term as in oncology this equates to five years observation period. At best, these studies represent a medium-term follow up window. Second, the control groups consisted of healthy individuals, but they did not have genetic testing for existing apo variants. Third, the observed individuals experienced acute infection that could be classified as septic shock, but definitions have varied over time and across studies. These studies were done before the modern understanding of how sepsis should be defined. Utilizing old definitions creates a significant problem for metanalyses. The studies were conducted by a small number of research groups without any subsequent investigation. The study’s population was also homogeneous and limited to a small geographical region.

To summarize, these studies suggested a shift in lipid profile as highlighted by a decrease in HDL-c and relative changes in LDL-c. Chemical modification of lipoproteins recovered to baseline during resolution of sepsis. VLDL-c and Lp(a) recovered as well. Changes in apoAI, apoB1, and ApoCIII seemed to be related to the aetiology of an infectious process. These processes have the potential ability to affect and respond to subsequent infection and accelerate atherosclerotic.

### 4.2. Clinical Implication of the Post-Sepsis Alterations in Lipid Profile

Only one study directly addressed clinical correlates of the post-sepsis altered lipid profile long-term. Kayner et al. follow up on mice surviving sepsis with autopsy at five months [192]. His finding demonstrated accelerated atherosclerosis in the ascending part of the aorta. These changes were linked to altered metabolism and are consistent with the emergence of post-sepsis inflammatory syndrome [20,46,193]. The authors specifically suggested that prolonged systemic endothelial and intimal inflammation was not related to accelerated atheroma. These findings suggested that lipid metabolism alterations, abnormalities on monocyte activation, or prolonged exposure to free radicals may be the underlying causes of accelerated atheroma formation in mice surviving sepsis. Only his study supports the former mechanism, but translation of his observation to humans may not be straightforward. The study was conducted in animals using the intra–abdominal models of sepsis in only male mice deficient in apoE [192]. Though the study was not replicated, it did cause some conversation in the critical care community, suggesting trained immunity as the mediator of accelerated atherosclerosis [55]. This idea is well aligned with the concept of post-septic changes in MO differentiation with the emergence of monocyte of M2 type in the aftermath of sepsis. Interestingly, the apolipoprotein imbalance may lead to the conversion of MO to macrophages or atypical monocytes. An increase in apoCI promotes activation of STAT3, potentially leading to the emergence of atypically activated macrophages, which are proatherogenic, while not responding to the infection as effectively as the M1 type [88].

### 4.3. Therapeutic Implication of the Post-Sepsis Alterations in Lipid Profile

If the abnormalities in the lipid profile persist for a protracted period, they may represent an attractive target for direct clinical intervention. In an animal model, supplementation of depleted HDL-c had a beneficial effect on the progression of atherosclerosis, but in genetically modified mice [194]. In addition, restoring hormonal balance, focusing on testosterone, may indirectly affect atherosclerosis progression post-sepsis [188].

Statins are traditionally used to modify lipid profiles. They also have an immunomodulatory and pleiotropic effect. However, their effectiveness post-sepsis is unknown and the data are somewhat conflicted [195]. Furthermore, statins seem to be under-prescribed in sepsis survivors in general [196]. One aspect that needs to be considered is that lipoproteins are severely modified during sepsis, while several lipoproteins elevated in sepsis (VLDL-c) are not susceptible to statin effects. Therefore, is it also possible that the effect of statin needs to be correlated to their pleiotropic effect on post-sepsis inflammation instead of classical interference with affecting HMG-CoA reductase [173,195].

Oxidation of LDL-c is one of the most potent triggers for atherosclerosis [56,125,126,127]. Free radical scavengers were utilized in several critical conditions, including sepsis, but few of these therapies demonstrated a beneficial effect in acute sepsis short term. The supplementation of free radical scavengers has poor evidentiary support in healthy individuals, and very little is known about effective ways to interrupt lipid oxidation in general [12,126,127,135]. The more suitable option may be an interference effect of LDL-c oxidation on their uptake by LOX-1. For example, migration of the monocyte into plaque is greatly enhanced by oxLDL-c after its uptake. Calpains are known as inhibitors of monocyte chemotaxis in response to LOX-1 binding [197]. Metformin and protamine demonstrate a similar effect, and they are already approved to use in humans to treat diabetes [198,199]. Some suggest utilization of PCSK9, considering their significant pleiotropic effect, but this is just a theory [200]. The potential effect of interfering with that pathway is a suppression of highly atherogenic Lp(a) [200].

One must remember that the treatment should be tailored to the post-septic lipid abnormalities and duration of the pro-atherogenic profile. Unfortunately, as of now, we do not know how long post-sepsis dyslipidaemia persists and how it evolves over time.

## 5. Potential Common Pathways between Lipoproteins Abnormalities and Sepsis Outcome in the Long-Term Perspective

Considering the pivotal structural role of cholesterol for cellular integrity, its role in steroid-type hormone synthesis, apoptosis, regulation of the immune response, and bile acid metabolism, it is not surprising that cholesterol abnormalities profoundly impact health maintenance. Any aberration in metabolism or cholesterol leads to a profound high-level impact, resulting in the acceleration of several degenerative processes [54,55,56,57,61]. On the other hand, significant depression of cholesterol impacts hormonal regulation and immune system function [201]. However, the excessive mortality at lower cholesterol serum levels could be the consequence of aetiology or premature demise.

Atherosclerosis has been linked to the progression of neurodegeneration, the decline in cardiac function, and end-organ failure. Dyslipidaemia, monocyte function, and endothelial damage are all implicated in the progression of atherosclerosis, yet it is unclear which of these three critical components would be most amenable and beneficial to manipulate to revert to the pre-sepsis homeostasis.

In this review, we purposely presented a very narrow focus on the lipid profile as the potential mediator of several long-term outcomes in sepsis only. The primary reason is that lipid metabolism is intertwined with metabolomic changes, dynamics of monocytes, endothelial dysfunction. These factors will interplay with potential abnormal profiles after sepsis, as suggested by relatively limited evidentiary support. It is unclear which one is more critical than others. It is also plausible that the co-existence of any of these factors together will lead to a synergistic effect on the acceleration of atherosclerosis.

It is possible that a similar condition exists in other illnesses related to atypical immune system activation, especially those related to chronic condition. Autoimmune disease, AIDS, or conditions after solid organ and bone transplant are examples of chronic inflammatory conditions. However, their pathology is further complicated by illness-specific immune system features. Consequently, they were not included in this review.

## 6. Summary

Acute sepsis is an extremely common affliction triggering profound changes in several key elements that lead to the progression of atherosclerosis in survivors. The data review shows a different effect on atherosclerosis progression during the acute vs. recovery phase of sepsis. During the acute phase, the lipid abnormalities are directly related to the survivorship of the affected individuals, while the long-term effect on atherosclerosis progression stem from the indirect effect of sepsis-associated hypolipoproteinaemia. Exaggerated inflammatory response, a hallmark of sepsis, may become even more pathological if LDL-c and HDL-c uncheck the initial inflammation. Other sepsis-related events mechanisms (MO and T cells reprogramming, metabolism changes, endothelial damage) may create pro-atherogenic features of a lipid profile in the acute phase and synergize the delivery of an acute insult to the endothelium, thus accelerating sepsis at that stage. Long-term persistence of a proatherogenic lipid profile seems to be evident in survivors, but a small number of studies limits generalization. The majority of these studies were underpowered. They lack analysis of genotype as the allele variants of apolipoproteins are a significant confounder [202,203]. Only one study directly linked the abnormalities in lipid profile to the acceleration of atheroma, but these data were obtained from animals exquisitely prone to accelerated atherosclerosis. The large metanalysis of predominantly acute lipid profile changes in sepsis demonstrated increased atherosclerosis-related mortality [47].

The potential implication that atherosclerosis can be accelerated in survivors of sepsis is important clinically and of far-reaching consequences for society. However, the clinical study directly addressing this issue is difficult to execute, and the paucity of data should be hardly surprising. Some of these limitations stem from the fact that sepsis is a heterogeneous disease in aetiology and individual response, introducing significant heterogeneity into the research and, therefore, difficulty to the interpretation of data [5,30,32,46]. Acquisition of patients for long-term studies is inherently difficult. Atherosclerosis depends on dietary influence, physical activity, individual resilience, genetic makeup, and lipid-modifying therapies. Incorporating these elements into prospective studies may be very challenging.

Atherosclerosis is a multifactorial process, and the lipid profile plays an important and complex role entangled in several other factors, including endothelial inflammation, monocyte differentiation, and free radical stress. Nevertheless, post-septic alterations of the lipid profile serve as attractive therapeutic targets considering several pharmacological options are already available.

## Figures and Tables

**Table 1:** Linkage of abnormalities in lipoproteins to emergence of pathological conditions.

Apo Type	Localization	Function	Role in Pathology
A1	HDL	Acceptance of fat from macrophages, prostacyclin stabilizing factor, lipopolysaccharide binding, neuronal regeneration [69]	Tangier disease, amyloidosis [44,70], cardiovascular disease progression [70,71], Alzheimer disease [72,73], sepsis [74], schizophrenia [75], chronic pain [76]
A2	HDL	Lipid transport, inflammation, neuronal regeneration [69]	Cardiovascular disease progression [43], amyloidosis [44,77]
A4	Chylomicrons, VLDL-C, HDL, hypothalamus	Appetite [78], antioxidants metabolism, lipid transport. Thermogenesis [79,80]	Schizophrenia [81]
A5(A-V)		Regulation of TG levels	Metabolic syndrome [82], cardiovascular disease progression [83]
B48	Chylomicrons, VLDL-C	Staphylococcal infection defence [49,51], weight loss, carbohydrate metabolism [84], Lipid transport [59]	Infection resistance [49,51], insulin resistance [84], venous thrombosis [85]
B100	Chylomicrons	Staphylococcal infection defence [51], Lipid transport [59]	Cardiovascular progression [59], insulin resistance, infection resistance [49,51], venous thrombosis [85]
C-I	HDL, VLDL-C, chylomicrons	Lipid metabolism [86]	Alzheimer’s disease [66,87], cancer promoting [88], venous thrombosis [85], pancreatitis, hepatosplenomegaly
C-II	VLDL-C, chylomicrons	Lipid metabolism [67,86]	Alzheimer’s disease [66,87]
C-III	VLDL-C, chylomicrons, remnant cholesterol, HDL	Lipid metabolism [68]	Alzheimer’s disease [66], coronary artery disease [68,89]
C-IV	Liver	Lipid metabolism [86]	Alzheimer’s disease [90], stroke [91]
D	Brain, testes, HDL	Lipoprotein metabolism [64], hormone binding [64], neuronal regeneration [69,92], inflammation [64]	AD [45], schizophrenia [93], Parkinson’s disease [94], multiple sclerosis [95], diabetes [64], cancer [64,96,97], multiple sclerosis [98], tangier disease, chronic pain [76], bone demineralization [99]
E	Chylomicrons remnants, VLDL-C, IDL, HDL, liver macrophages, astrocytes,	Lipid metabolism [86], neuronal regeneration [69,92], complement activation, vitamins transport, immunosuppression [100]	Cardiovascular disease [71,101], Alzheimer’s disease [66,87,102,103,104], stroke [91], neurodegeneration [105,106], traumatic brain injury [107]
F	HDL	Lipoprotein metabolism [65]	Schizophrenia [81], stroke [91]
β2-glycoprotein 1 (H)	platelets	Platelets function [108]	Lupus [109]
L	Kidney, adipoase tissue	Triggering programmed death [110]	Schizophrenia [111]; kidney disease [112], trypanosomes [112], stroke [91]
M	VLDL-C, HDL, LDL-C	Lipid metabolism [52], carbohydrate metabolism [113,114]	Metabolic syndrome [113], cancer [115], cardiovascular disease [114,116]
(a)	Bound to ApoB	Lipid metabolism [117,118], thrombogenesis [48,53], inflammatory response [50]	Stroke [48,53], coronary heart disease [119]

## Data Availability

Not available.
